# Calciphylaxis as a Rare Complication Associated with Pemigatinib Treatment—A Case Report

**DOI:** 10.3390/curroncol33060360

**Published:** 2026-06-15

**Authors:** Katarina Čular, Dora Tomek Hamzić, Ljiljana Smiljanić Tomičević, Daška Štulhofer Buzina, Mirna Bradamante, Luka Simetić, Ivan Bilić, Borislav Belev

**Affiliations:** 1 Department of Oncology, University Hospital Center Zagreb, 10000 Zagreb, Croatia; katarina.cular@kbc-zagreb.hr (K.Č.); lsimetic@kbc-zagreb.hr (L.S.); ivan.bilic@kbc-zagreb.hr (I.B.); 2 Division of Clinical immunology and Reumathology, Department of Internal Medicine, University Hospital Center Zagreb, 10000 Zagreb, Croatia; ljiljana.smiljanic.tomicevic@kbc-zagreb.hr; 3 Department of Dermatovenerology, University Hospital Center Zagreb, 10000 Zagreb, Croatia; daska.stulhofer-buzina@kbc-zagreb.hr (D.Š.B.); mirna.bradamante@kbc-zagreb.hr (M.B.); 4 School of Medicine, University of Zagreb, 10000 Zagreb, Croatia

**Keywords:** pemigatinib, calciphylaxis, cholangiocarcinoma

## Abstract

Fibroblast growth factor receptor 2 (FGFR2) inhibitors such as pemigatinib are targeted therapies for cholangiocarcinoma with FGFR2 alterations. While generally well tolerated, they are associated with unique adverse events. Calciphylaxis, a potentially fatal vascular calcification disorder, is a rare complication. We present a 43-year-old woman with metastatic intrahepatic cholangiocarcinoma harboring an FGFR2 fusion who developed calciphylaxis after seven months of pemigatinib therapy. Despite drug discontinuation, antibiotics, and multidisciplinary supportive care, she deteriorated rapidly and died from sepsis and advanced disease. Histopathological analysis confirmed dermal and vascular calcifications consistent with calciphylaxis. This case highlights the importance of early recognition of cutaneous lesions in patients on FGFR inhibitors. Prompt cessation of therapy, management of metabolic derangements, and consideration of sodium thiosulfate may be lifesaving.

## Simple Summary

Pemigatinib, an FGFR2 inhibitor, is used to treat FGFR2-altered cholangiocarcinoma but may lead to rare, serious side effects. We report a 43-year-old woman with metastatic intrahepatic cholangiocarcinoma who developed calciphylaxis—a rare, life-threatening condition involving vascular calcification—after seven months of pemigatinib. Despite stopping the drug and initiating antibiotics and supportive care, her condition worsened, and she died from sepsis and disease progression. Skin biopsy confirmed calciphylaxis. This case highlights the importance of recognizing skin changes in patients treated with FGFR inhibitors at an early stage. Stopping therapy and managing complications promptly may improve outcomes.

## 1. Introduction

Fibroblast growth factor receptor 2 (FGFR2) inhibitors, recently in clinical use, including pemigatinib, futibatinib, and infigratinib, are novel therapies for cancers driven by FGFR2 fusions, mutations, or rearrangements. FGFR2 is a receptor tyrosine kinase regulating cell differentiation, proliferation, angiogenesis, and tissue repair. Aberrant FGFR2 signaling drives oncogenesis via MAPK, PI3K/AKT, and STAT pathways and is enriched in cholangiocarcinoma (CCA), accounting for ~7% of cases [1]. FGFR inhibitors selectively block aberrant signaling, suppressing tumor progression. Pemigatinib has received approval as second- or third-line therapy for metastatic iCCA with FGFR2 fusions, based on the FIGHT-202 trial [2].

Like other targeted therapies, FGFR2 inhibitors are associated with a unique toxicity profile. Common adverse effects with pemigatinib include electrolyte disturbances (notably hyperphosphatemia and hyponatremia), diarrhea, fatigue, alopecia, and various cutaneous, ocular, and nail toxicities. While most side effects are manageable with supportive care and dose modifications, rare but serious complications can occur. Among these is calciphylaxis, a potentially life-threatening condition characterized by calcification of small blood vessels, leading to ischemia, tissue necrosis, and painful skin lesions.

This case report describes a patient who developed calciphylaxis during pemigatinib treatment. We outline the clinical course, diagnostic challenges, potential underlying mechanisms, and therapeutic considerations in the context of existing literature.

## 2. Case Report

In the summer of 2023, a 43-year-old female patient, otherwise healthy, presented to our institution with metastatic cholangiocarcinoma. She had initially been diagnosed in October 2021 with locally advanced intrahepatic cholangiocarcinoma primarily affecting the right lobe and treated at another center. First, she underwent transarterial chemoembolization (TACE) of the right hepatic artery. Afterwards, she received stereotactic body radiotherapy (SBRT) with a total dose of 25 Gy to the right lobe lesion. Subsequently, atypical resection of segments V–VII with additional metastasectomy of the lesion in segment VII was performed. Histopathology confirmed poorly differentiated adenocarcinoma consistent with intrahepatic cholangiocarcinoma, classified as pT2N1M1 with positive resection margins.

Through 2023, she received four additional SBRT sessions and four cycles of combined chemoimmunotherapy with gemcitabine, cisplatin, and durvalumab. Further details of these treatments were unavailable, as they had been undertaken at another institution. In August 2023, she presented to our center with lung and liver metastases. Next-generation sequencing (NGS) of tumor tissue revealed an FGFR2 fusion, and in October 2023, she commenced treatment with pemigatinib.

The patient started on oral pemigatinib (Pemazyre) 13.5 mg daily, given for 14 days followed by a 7-day pause. Initially, the treatment was well tolerated, with only mild adverse effects including fatigue, arthralgia, mucositis, nail changes, alopecia, and dry skin, none of which required dose modification. Imaging after three and six months showed a favorable response.

However, after seven months of therapy, she developed a small erythematous skin lesion with an underlying bulla under the right breast. Pemigatinib was continued for two additional cycles, as the lesions initially appeared mild and non-progressive. By the end of May 2024, when the last cycle was initiated, the lesions had begun to increase in size and number, evolving from erythematous to bullous and eventually to large areas of necrosis.

The patient sought care at another institution, where toxic epidermal necrolysis (TEN, Lyell syndrome) was suspected. Management included wound debridement, dressings, and supportive care. Biopsy at that time reported necrotic adipose and connective tissue with calcifications.

**Figure 1:**
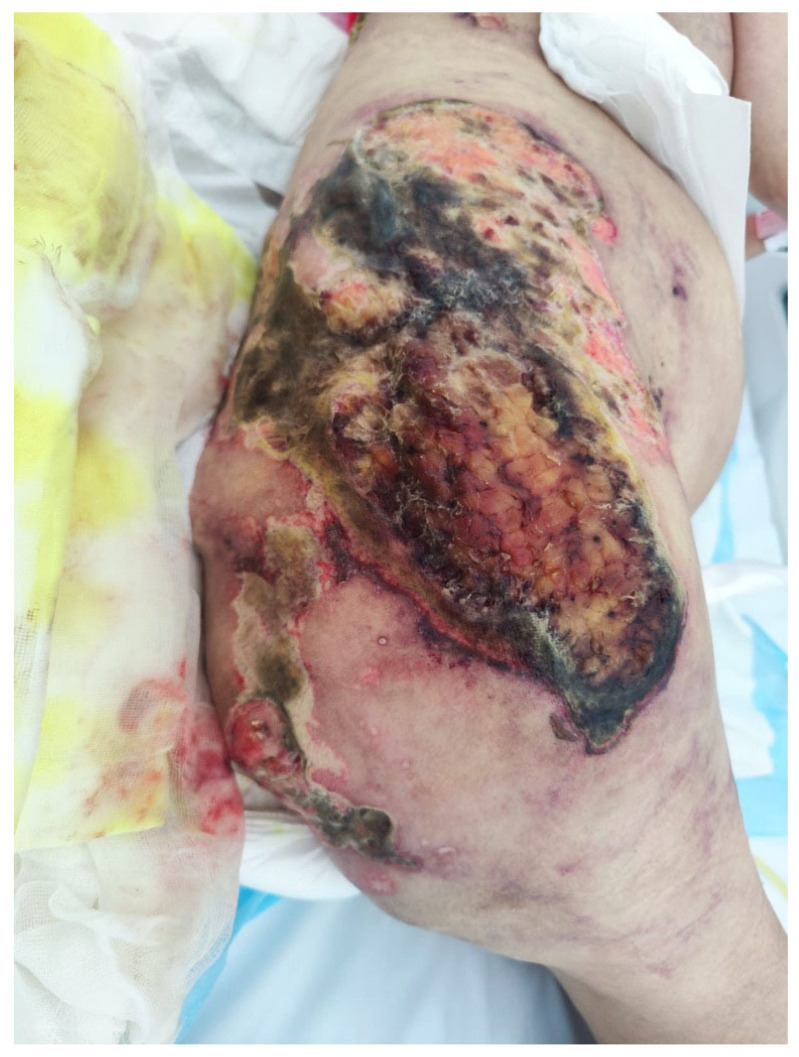
Extensive necrotic skin lesions.

She was re-admitted to our institution in July 2024 with somnolence, sepsis, and extensive necrotic skin lesions up to 2 cm in depth and 15 cm in diameter, affecting more than 30% of her body surface area (Figure 1). Laboratory results revealed microcytic anemia, leukocytosis, elevated C-reactive protein (197 mg/L; reference < 5), elevated procalcitonin (3.73 µg/L; reference < 0.25), impaired renal function (Cockcroft-Gault eGFR 28 mL/min; reference > 90), cholestatic liver dysfunction, hypoalbuminemia, hypomagnesemia, and mild hyperphosphatemia (1.66 mmol/L; reference 0.79–1.42) with normocalcemia (total calcium 2.38 mmol/L; ionized calcium 1.26 mmol/L).

**Figure 2:**
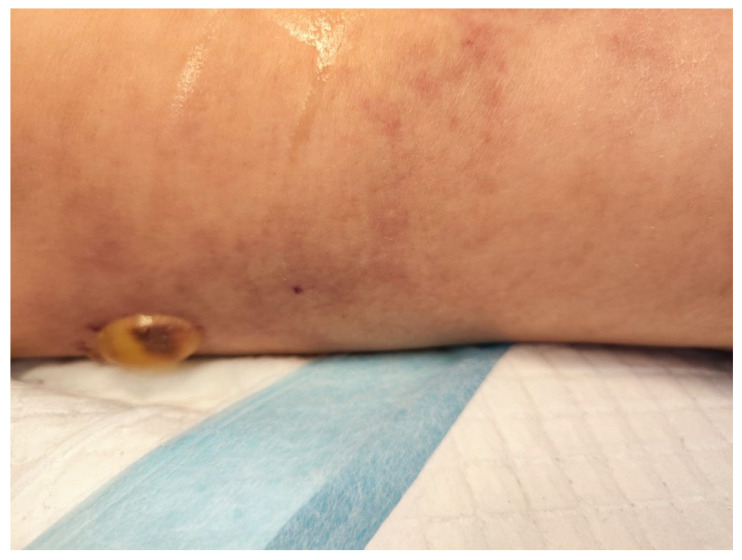
Bullous lesions on the left thigh, from which a biopsy was obtained.

**Figure 3:**
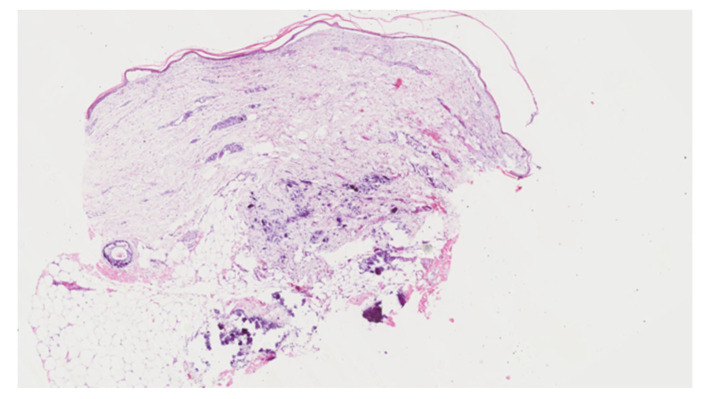
HEx10: Calcium deposits were seen in the dermis and subcutis, and within the vessel wall.

Wound cultures grew multiple Gram-positive and Gram-negative bacteria, including *Enterobacter hormaechei*, *Enterococcus faecalis*, *Stenotrophomonas maltophilia*, *Pseudomonas aeruginosa*, *Escherichia coli*, *Staphylococcus aureus*, and *Peptoniphilus* spp. She was treated with broad-spectrum intravenous antibiotics (vancomycin and meropenem), analgesics, intravenous fluids with albumin supplementation, and loop diuretics. Local wound care was continued. Because of the complexity of the case, the dermatology, plastic surgery, and immunology teams were consulted. Biopsies of two skin lesions were performed under the clinical suspicion of a drug reaction. Within the dermis and subcutaneous fat in both of them calcium deposits were seen. In a biopsy from a thigh (Figure 2), deposits were also appreciated within the vessel wall in the subcutaneous fat, prompting the diagnosis of calcifilaxis (Figure 3).

**Figure 4:**
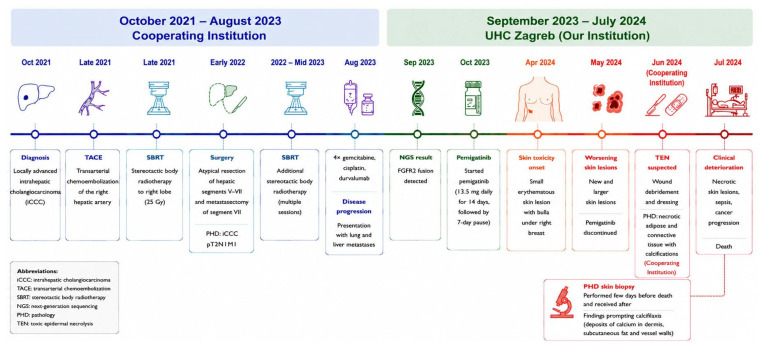
Course of disease *. * This illustration was created with the assistance of artificial intelligence using ChatGPT Plus Version 2.0.

Despite intensive management, the patient’s condition continued to deteriorate, and she died of sepsis and terminal malignant disease soon after the biopsy, before the pathohistological results became available (Figure 4).

## 3. Discussion

Cholangiocarcinoma (CCA) is a rare cancer, accounting for less than 1% of all human cancers. The term biliary tract cancer (BTC) encompasses intrahepatic cholangiocarcinoma (iCCA), extrahepatic cholangiocarcinoma, and gallbladder carcinoma [3].

Localized CCA is potentially curable with surgery; however the high relapse risk necessitates adjuvant chemotherapy or chemoradiotherapy regardless of pathological stage, as per European (ESMO) and American (NCCN) guidelines. Advanced or metastatic CCA is generally incurable and associated with a poor prognosis. Currently, first-line therapy is chemoimmunotherapy with durvalumab, gemcitabine, and cisplatin, while second-line options include fluorouracil- and oxaliplatin-based chemotherapy or targeted therapy when actionable mutations are identified.

Next-generation sequencing is strongly recommended in BTCs because they display diverse molecular profiles and 40–50% of patients harbor targetable mutations, with *IDH* and *FGFR2* mutations being the most frequent [3].

*FGFR2* fusions and rearrangements are driver mutations in approximately 7% of CCAs [1]. The FGFR family (FGFR1-4) comprises transmembrane receptor tyrosine kinases essential for organogenesis, tissue regeneration, and cell proliferation. Activating *FGFR* mutations produce aberrantly active proteins promoting uncontrolled cell growth and tumorigenesis [4].

FGFR inhibitors (pemigatinib, futibatinib, infigratinib) are small-molecule agents that inhibit FGFR 1/2/3 phosphorylation, reducing the viability of mutated cancer cells [1]. Phase II clinical trials demonstrated response rates of 20–40%, median progression-free survival of 7 months, and overall survival of 12–17 months in pretreated FGFR2-mutated CCA patients [3]. The FIGHT-202 trial led to FDA and EMA approval of pemigatinib for second- and third-line treatment of FGFR2-mutated advanced CCA [2].

Known side effects of pemigatinib include electrolyte imbalances (hyperphosphatemia, hypophosphatemia, hypercalcemia), alopecia, diarrhea, nail toxicity, fatigue, nausea, stomatitis, dry mouth and skin, and eye disorders (including rare serous retinal detachment), among others [5]. Skin and subcutaneous adverse events are common: alopecia and nail changes affect ~50% of patients; dry skin affects ~20%, while palmar-plantar erythrodysesthesia and abnormal hair growth are less frequent [5]. Calciphylaxis, characterized by cutaneous calcification, is a rare but serious adverse effect associated with FGFR inhibitors.

Calciphylaxis is characterized by calcification of small- to medium-sized cutaneous vessels, tissue ischemia, and necrosis, most frequently described in patients with end-stage renal disease or severe disturbances in calcium–phosphate metabolism. Nevertheless, the pathophysiology of calciphylaxis is obviously very complex and different conditions may be contributing factors—such as diabetes, hypercoagulable state, hypercalcemia and hyperphosphatemia, and inflammation.

The condition typically presents with painful plaques or ulcerations that may rapidly progress to necrosis, similar to this case. This patient initially presented with bullous lesions, which subsequently progressed to necrosis. It carries a poor prognosis with a high one-year mortality [6]. The patient developed widespread skin involvement after seven months of pemigatinib therapy, ultimately complicated by sepsis and death.

Complications of calciphylaxis range from moderate interference with activity to death. Lesions of calciphylaxis frequently result in nonhealing ulcers and cutaneous gangrene. Acral lesions may fail to heal with conservative therapy and lead to amputation. Sepsis may result from the nonhealing wounds. Patients with internal involvement may develop gastrointestinal hemorrhage, infarction, or organ failure [7].

In a literature search using PubMed and Google Scholar with “pemigatinib AND calciphylaxis,” we identified six reported clinical cases describing calciphylaxis in patients treated with pemigatinib [4,8,9,10,11,12] and one case linked to futibatinib [13]. These cases discuss possible pathophysiology and management strategies. Although definitive mechanisms remain unclear, several contributing factors may be considered. First, hyperphosphatemia related to FGFR inhibition may play a role, as FGFR1 normally regulates phosphate homeostasis via fibroblast growth factor 23 (FGF23) signaling. Inhibition by FGFR-targeted agents may reduce renal phosphate excretion, leading to persistently elevated serum phosphate levels. Although our patient exhibited only mild hyperphosphatemia, chronic disturbances may have contributed to vascular calcification. Furthermore, FGFR signaling is involved in vascular integrity and endothelial repair; its inhibition may predispose to vascular injury and promote calcification. Finally, concomitant factors such as hypoalbuminemia, renal impairment, and advanced malignancy likely increased susceptibility in this patient. The presence of several of these risk factors at presentation may have acted synergistically with pemigatinib exposure.

Differential diagnosis was challenging. The lesions were initially mistaken for toxic epidermal necrolysis, underscoring the potential for misdiagnosis given the overlapping clinical features of widespread necrosis and ulceration. The differential diagnosis should include various conditions, such as bullous pemphigoid, bullous systemic lupus erythematosus (SLE), erythema nodosum, hypersensitivity vasculitis, pyoderma gangrenosum and others. Histopathological confirmation remains the diagnostic gold standard. However, in this case, the definitive biopsy results indicating dermal and vascular calcification became available only a few days after death, as the patient died of sepsis and terminal malignant disease less than 2 days after skin biopsy.

Management of calciphylaxis is notoriously difficult and not standardized. It includes withdrawal of the offending agent, correction of metabolic derangements (especially hyperphosphatemia and hypercalcemia), wound care, and infection control. Generally, mild hyperphosphatemia is managed with dietary phosphate restriction and oral phosphate binders (e.g., sevelamer). Discontinuation of pemigatinib is recommended when hyperphosphatemia persists or calciphylaxis signs develop. Sodium thiosulfate, administered topically or intravenously two to three times weekly, is commonly used off-label due to its calcium solubilizing, anti-inflammatory, antioxidant, and vasodilatory properties [14]. Despite broad-spectrum antibiotics, supportive care, and discontinuation of pemigatinib, the patient developed fulminant sepsis and succumbed to her illness, reflecting the high mortality rates reported in calciphylaxis, often exceeding 50%. In our patient, sodium thiosulfate was not administered due to delayed biopsy results and phosphate binders could not be given because of somnolence impairing oral intake.

Of the seven cases identified, five achieved successful resolution of calciphylaxis lesions with these treatment modalities. Prognosis depends on the extent of the skin lesions, the underlying disease burden, and the overall performance status. None of the cases resumed FGFR inhibitor therapy after lesion resolution.

## 4. Conclusions

Calciphylaxis is a rare but severe adverse event associated with FGFR inhibitors, often carrying a poor prognosis. Early recognition and multidisciplinary management are paramount, including prompt cessation of pemigatinib, correction of phosphate and calcium abnormalities, and consideration of sodium thiosulfate alongside supportive wound care.

This case underscores the need for early recognition of cutaneous lesions arising in patients treated with FGFR inhibitors. Even seemingly mild skin changes should prompt careful investigation, including consideration of calciphylaxis. Regular monitoring of phosphate, calcium, albumin, and renal function during pemigatinib therapy is essential to anticipate and mitigate risks. Reporting such cases contributes to a better understanding of the full toxicity spectrum of FGFR2 inhibitors and may lead to earlier recognition and intervention in future patients.

## Data Availability

The data presented in the study are quite relevant and additional data are available on request from the corresponding authors due to privacy reasons.
